# Molecular Cytogenetic Characterization of Novel 1RS.1BL Translocation and Complex Chromosome Translocation Lines with Stripe Rust Resistance

**DOI:** 10.3390/ijms23052731

**Published:** 2022-03-01

**Authors:** Zhi Li, Zhenglong Ren, Feiquan Tan, Peigao Luo, Tianheng Ren

**Affiliations:** 1 Agronomy College, Sichuan Agricultural University, Chengdu 611130, China; lizhi@sicau.edu.cn (Z.L.); renzllab@sicau.edu.cn (Z.R.); feiquantan_1@163.com (F.T.); lpglab@sicau.edu.cn (P.L.); 2 Provincial Key Laboratory for Plant Genetics and Breeding, Sichuan Agricultural University, Chengdu 611130, China

**Keywords:** wheat, rye, 1RS.1BL translocation, complex chromosome translocations, stripe rust, FISH, genetic resource

## Abstract

Rye is the most important source for the genetic improvement of wheat. In this study, two stable wheat-rye primary 1RS.1BL translocation lines, RT855-13 and RT855-14, were selected and identified by acid polyacrylamide gel electrophoresis (A-PAGE), co-dominant PCR, and multi-color fluorescence in situ hybridization (MC-FISH) from the progeny of the crossing of the wheat cultivar Mianyang11 and a Chinese rye Weining. When more than two independent, simple reciprocal translocations are involved in a carrier, they are defined as complex chromosome translocations (CCT). The MC-FISH results also indicated that CCT occurred in RT855-13; namely that, besides 1RS.1BL translocation chromosomes, there are other two pairs of balanced reciprocal translocations. It was demonstrated that the interchange between a distal segment of 4B and long arm of 3D occurred in the RT855-13. The novel translocation chromosomes in wheat were recorded as 3DS.4BS^DS^ and 3DL-4BS^PS^.4BL. Reports about CCT as a genetic resource in plant breeding programs are scarce. Both lines expressed high resistance to *Puccinia striiformis* f. sp. *tritici,* which are prevalent in China and are virulent on *Yr9*, and the CCT line RT855-13 retained better resistance as adult plants compared with RT855-14 in the field. Both lines, especially the CCT line RT855-13, exhibited better agronomic traits than their wheat parent, Mianyang11, indicating that both translocation lines could potentially be used for wheat improvement. The results also indicated that the position effects of CCT can lead to beneficial variations in agronomic and resistant traits, making them a valuable genetic resource to wheat breeding programs.

## 1. Introduction

Wheat (*Triticum aestivum*) is an allohexaploid crop containing three genomes (2n = 6× = 42, AABBDD). The tetraploid *T. turgidum* (2n = 4× = 28, AABB) was first originated from the progeny of the polyploidization of *T. urartu* (donor of A genome) and an unknown diploid species of the *Sitopsis* (donor of B genome) [1]. Then, the hexaploid wheat was generated from the allopolyploid between *T. turdidum* and *Aegilops tauschii* (donor of D genome) about 10,000 years ago [1,2]. Numerous reciprocal or Robertsonian translocations have been detected in global accessions of polyploidy wheat species [3,4,5]. Translocations between B-genome chromosomes in wheat are found more frequently than the A and D genome chromosomes, and chromosomes 1A, 4A, 5A, 2D, 5D, 5B, 6B, and 7B were more frequently involved in translocations than other chromosomes [5,6]. In addition to intra-species chromosomal translocations, alien chromosome translocations have played important roles in wheat-breeding programs worldwide, resulting in the development of excellent traits and enhanced the genetic diversity of common wheat, which have been widely utilized in wheat-breeding programs [2,7,8,9,10,11,12,13,14,15]. Some wheat genotypes with translocation chromosomes exhibit more attractive agronomic traits, such as resistance to diseases or other adaptive advantages that may have led to their distribution in diverse eco-geographical regions [5,16,17]. Rye (*Secale cereal* L.) is the most useful related species for wheat genetic improvement because of its advantages compared with others [18,19,20]. Many useful genes, such as *Yr9* (stripe rust), *Pm8* (powdery mildew), *Lr26* (leaf rust), *Sr31* (stem rust), and several important factors, such as yield-enhanced factors and the environmental adaptability factors, were transferred into wheat through 1RS.1BL translocation involving the short arm of chromosome 1 of rye, 1RS, and the long arm of chromosome 1 of wheat, 1BL, in the 1950s [20,21,22,23,24]. This translocation chromosome was used widely in wheat-breeding programs [19]. Unfortunately, due to its single origin, the *Yr9* gene has not provided protection against stripe rust (caused by *Puccinia striiformis* f. sp. *tritici*, *Pst*) since the 1990s [23,25].

Identification and confirmation of translocations are difficult and time-consuming; using conventional cytogenetic techniques [26], fluorescent in situ hybridization (FISH), and molecular techniques have made this process easier, more reliable, and less costly [27,28,29,30]. Such techniques have been employed to effectively identify newly generated translocations between wheat and its relatives [28,29,30,31].

When a genotype contains two or more independent, simple reciprocal translocations, these translocations are defined as complex chromosome translocations (CCT). Numerous studies in human genetics have reported that CCT could acutely or chronically lead to fast aging, genetic disorders, and cancers [32]. Polyploidy plants, such as wheat, can tolerate chromosome abnormalities better than diploids. However, so far, there are limited reports about the development and identification of CCT in the plant. The mechanism that generates CCT is unclear, and the assessment of CCT as a genetic resource in plant breeding remains scarce. In this paper, we report a novel CCT wheat germplasm that was developed by inducing multiple translocations involving 1B, 1R, 4B, and 3D chromosomes of wheat and rye, and this CCT line showed high resistance to stripe rust and excellent grain yield.

## 2. Results

### 2.1. Development and Identification of the Novel Translocation Lines

**Figure 1:**
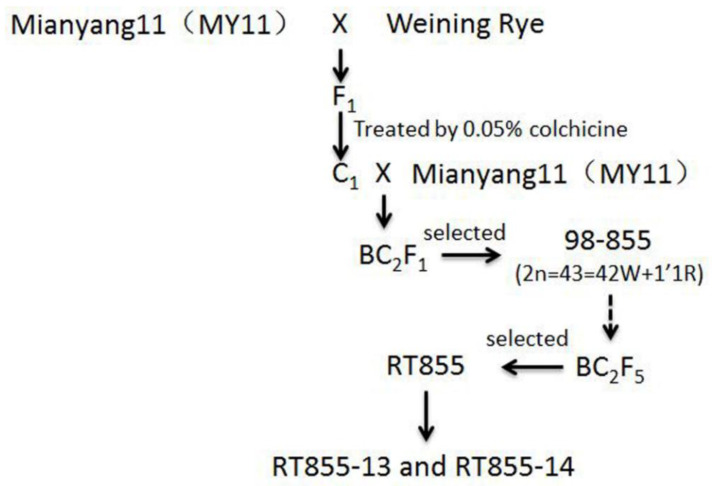
The pedigree of new translocation lines.

The pedigree of RT855-13 and RT855-14 is displayed in Figure 1. From the progenies of RT855 (2n = 41), two novel translocation lines, RT855-13 and RT855-14, were selected. All of the progeny of RT855-13 and RT855-14 displayed similar morphological characters, respectively, and were considered new primary 1RS.1BL translocation lines. A-PAGE, codominant PCR, and MC-FISH were used to identify the chromosome structure of RT855-13 and RT855-14.

**Figure 2:**
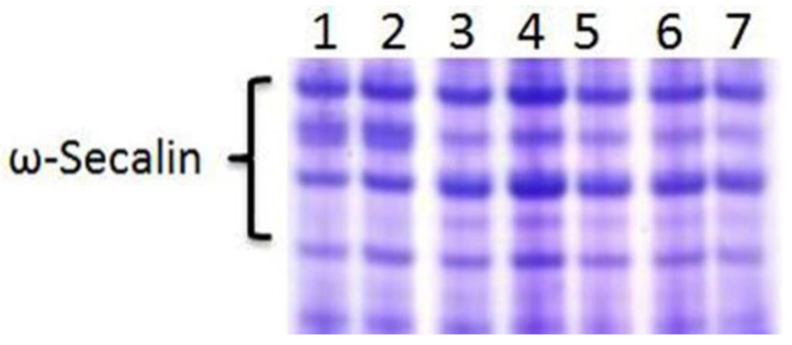
Acid polyacrylamide gel electrophoresis (A-PAGE) patterns of ω-secalins and gliadins from RT855-13 and RT855-14. Lane 1–3, RT855-13 from different generations; lane 4–6, RT855-14 from different generations; lane 7, CN11 (control).

The ω-secalin protein was encoded by the *Sec-1* gene on the 1RS chromosome arm of rye, with a specific electrophoretic pattern in wheat genetic background. The results showed that all RT855-13 and RT855-14 samples exhibited the expression of the *Sec-1* gene (Figure 2). It was indicated that RT855-13 and RT855-14 contained the 1RS chromosome arm, which was derived from Weining rye.

**Figure 3:**
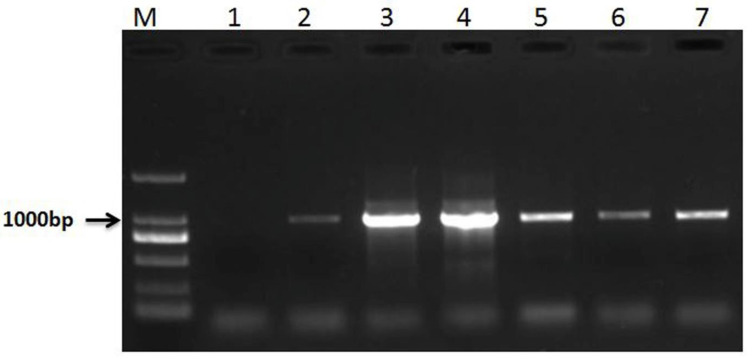
Results of co-dominant PCR. Lane 1, water (negative control); lane 2–4, PCR results of RT855-13; lane 5–7, PCR results of RT855-14; lane M, marker.

Co-dominant PCR markers were used to identify RT855-13 and RT855-14. A 630-bp band of 1BS was amplified by primer pair O11B3 and O11B5, and a 1076-bp band of 1RS was amplified by primer pair ω-sec-P1 and ω-sec-P2. When these two primer pairs are used together in one PCR reaction, the homozygous 1RS.1BL translocation lines could amplify a 1076-bp band but not result in 630-bp band amplification. The co-dominant PCR results showed that RT855-13 and RT855-14 can amplify a 1076-bp band but not a 630-bp band (Figure 3). It was indicated that both lines were homozygous 1RS.1BL translocation lines.

**Figure 4:**
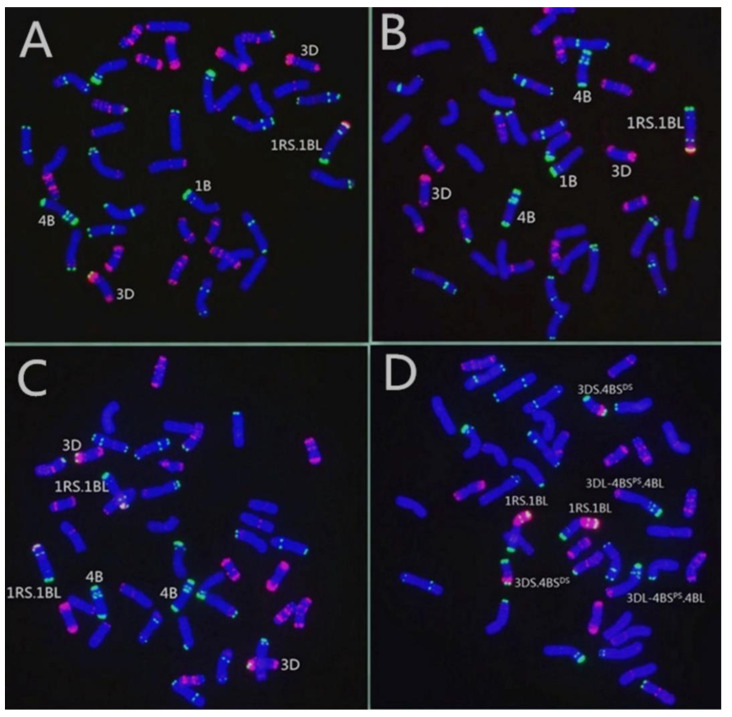
Developing process of the novel translocation lines harboring 1RS.1BL and balanced complex reciprocal translocations. (**A**) RT855 (2n = 41); a plant was selected from BC_2_F_5_, with a new alien translocation 1RS.1BL and deleting a 4B; note that a pair of normal 3D chromosomes are intact. (**B**) A plant selected from progenies of RT855, 2n = 42, 4B obtained correctly. (**C**) A novel primary 1RS.1BL translocation line, RT855-14 (2n = 42), derived from RT855, in which 4B and 3D are normal. (**D**) A pure, balanced CCT line, RT855-13 (2n = 42), derived from RT855, harboring three pairs of translocation chromosomes. Translocation between 3D and 4B chromosomes was generated, accompanying 1R/1B alien translocation and a 4B deletion.

**Figure 5:**
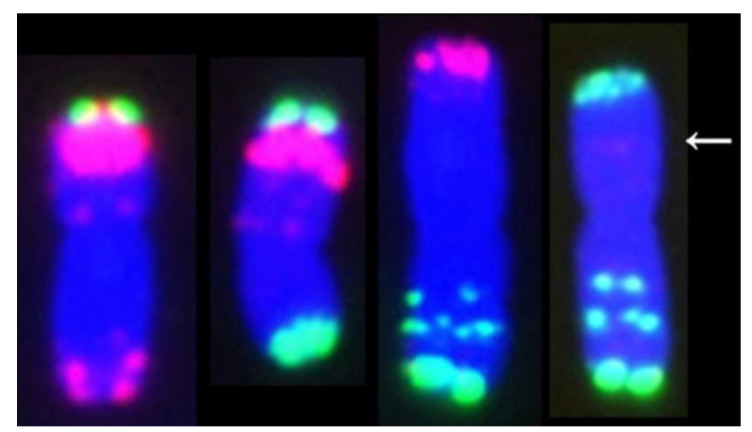
A comparison of chromosomes containing translocation and normal chromosomes. From left to right: 1, normal 3D; 2, 3DS.4BS^DS^; 3, 3DL-4BS^PS^.4BL; 4, normal 4B. In the CCT line, the novel chromosome 3DL-4BS^PS^.4BL became one of the largest chromosomes, and the 3DS.4BS^DS^ is one of the smallest chromosomes of wheat. The arrow indicates a possible breakpoint.

Then, both translocation lines were examined by MC-FISH. The results of MC-FISH showed that RT855-14 (2n = 42) contains an intact pair of wheat-rye 1RS.1BL translocation chromosomes (Figure 4C). However, regarding another translocation line, RT855-13, we determined that it is a CCT line and harbored three pairs of translocation chromosomes, a pair of 1RS.1BL chromosomes, and balanced reciprocal translocations between 4B and 3D chromosomes of wheat (Figure 4D). We determined that accompanying the 4B deletion in the plant, the 3D and 4B chromosomes of wheat generated rearrangements. A rearranged chromosome in the wheat line, recorded as 3DS.4BS^DS^, consisted of a whole arm of 3DS and a distal segment of 4BS. In the corresponding rearranged chromosome, the original 4B chromosome had lost a distal segment from the short arm and received a whole 3DL. The novel chromosome consisted of the 3DL, a proximal segment of 4BS, and the 4BL (Figure 5), recorded as 3DL-4BS^PS^.4BL.

**Figure 6:**
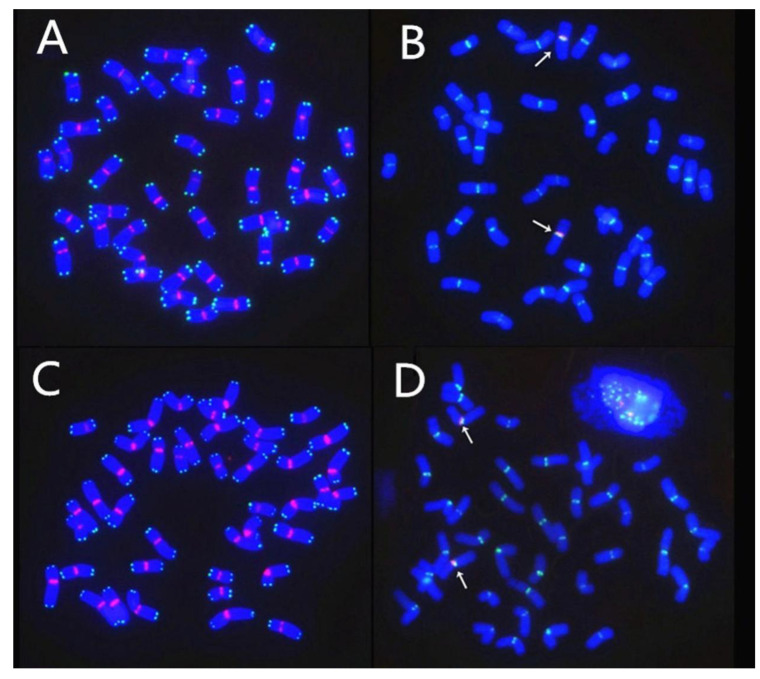
Fluorescent in situ hybridization (FISH) analysis of centromeres and telomeres in chromosomes containing simple and complex translocations. All chromosomes were shown to be intact. (**A**,**B**) The line RT855-14 with simple chromosomes translocation. (**C**,**D**) The line RT855-13 with CCT. (**A**,**C**) The signals of clone 6c6 for centromeres (red) and of telomere probe (green) indicated that all chromosomes in both translocation lines possess normal centromere and telomere. (**B**,**D**) The signals of clone 6c6 for wheat centromeres are green, and signals (red) of rye-specific centromere sequence clone pMD-CEN-3 (arrows) indicated that alien translocation lines RT855-13 and RT855-14 involved a same arm of 1RS from Weining rye.

MC-FISH analysis also demonstrated that all chromosomes of both translocation lines RT855-13 and RT855-14 contain normal centromeres and telomeres (Figure 6A,C). Rye-specific centromere sequence clone pMD-CEN-3 (Figure 6B,D) indicated that both translocation lines involved an intact arm of 1RS, which originated from the same 1R chromosome of the rye variety ‘Weining’.

### 2.2. Effect of Translocations on Agronomic Traits

Translocation lines RT855-13 and RT855-14 were derived from the same plant RT855 (2n = 41) (Figure 4A), which originated from the pure wheat line MY11 and a 1R chromosome. Therefore, both lines have the same genetic material from wheat and rye. Thus, any differences between the translocation lines RT855-13 and RT855-14 should primarily be in the effects of CCT.

Compared with their wheat parent MY11, both translocation lines exhibited slenderer leaves, flexible stalks, and tight plant type. Significant differences (*p* < 0.05) between the translocation lines and wheat parent MY11 were observed for kernel weight per spike (KW), 1000-kernel weight (TKW), number of spikes per square meter (NS), and grain yield (GY) (Table 1). For both lines, significantly lower KW was significantly correlated with lower TKW. However, compared with their wheat parent, these lines had significantly more NS, which led to a substantially higher grain-yield potential (Table 1). These results indicate there are positive effects of 1RS.1BL translocation on some agronomic traits and growth habits of wheat. Moreover, the CCT line RT855-13, which harbors three pairs of translocation chromosomes, grew well in the field, which is expected because it has balanced reciprocal translocation chromosomes. This line has a shorter stalk, larger spike, more prostrate growth in the winter, and later heading stage comp than RT855-14. Significant differences (*p* < 0.05) were observed only between the CCT line RT855-13 and MY11 for plant height (PH), spikelet number per spike (SLN), kernel number per spike (KN), and harvest index (HI) (Table 1). Additionally, significant differences (*p* < 0.05) between the RT855-13 and RT855-14 were observed for PH and SLN. These results indicate there are positive effects of CCT on some agronomic traits and growth habits of wheat.

### 2.3. Resistance Analysis of Translocation Lines

Wheat parent MY11 was susceptible to four *Pst* isolates, while Weining rye was resistant to four *Pst* isolates (Table 2). Wheat cultivar CN11, in which 1RS chromosome arm was derived from Petkus rye (*Yr9*), was highly susceptible to CYR29, CYR31, and SY5 (Table 2). When inoculated with *Pst* isolates in the greenhouse, the two translocation lines, RT855-13 and RT855-14, showed better resistance to stripe rust than MY11 and CN11 (Table 2). These results suggested that both translocation lines inherited new resistance genes from rye parent Weining by introducing the 1RS chromosome arm. However, in the field, the CCT line RT855-13 showed significantly better resistance to stripe rust as an adult plant than the 1RS.1BL translocation line T855-14 (Table 2). Because the lines RT855-13 and RT855-14 are derived from the same wheat parent plant, we assume differences in resistance to stripe rust result from different translocation events.

## 3. Discussion

### 3.1. Origin of Complex Chromosome Translocations

In humans, CCT is very harmful, leading to cancer and other lethal genetic disorders [33]. Therefore, in most cases, the CCT in humans could not be maintained in a population. However, polyploid plants, differing from human and other diploid plants, can tolerate more chromosome aberrations. It is documented that chromosomal translocations are important driving forces in the genomic evolution of wheat [2,5,34,35]. Research on artificially induced CCT would be useful not only for understanding complex genome structure alterations in the evolution of wheat but also for wheat improvement. In the present study, a CCT line was artificially induced from plants with a known pedigree (Figure 1): an aneuploid plant, 2n = 41, with a new 1RS.1BL translocation chromosome and deleted a 4B chromosome. This aneuploid plant was selected from a BC_2_F_5_ population, which was created through manipulation of a population derived from the monosomic addition line of the 1R chromosome. The novel line with CCT involved a pair of alien translocations between chromosomes1B and 1R of wheat and rye and two pairs of reciprocal interchanges between the long arm of 3D and a distal segment of 4BS (Figure 5). The novel rearranged chromosomes are named 3DS.4BS^DS^ and 3DL-4BS^PS^.4BL.

Numerous studies have documented that chromosomal abnormalities happen during periods of chromosome-genome instability in cells [32], which can be used as an approach to generate alien translocations that are desirable in wheat breeding. Several strategies have been used to induce chromosome-genome instability and transfer alien segments into wheat. Through inducing chromosome instability by ionizing radiation, Sears [36] transferred a leaf rust-resistance gene (*Lr9*) from *Ae. umbellulata* to wheat. Other methods for inducing chromosome instability are exploitation of gametocidal genes [37,38] and utilization of mutants or null alleles of *Ph1* gene, which induce homoeologous chromosome pairing between wheat and the alien chromosomes [36]. In aneuploid plants, such as chromosome addition and substitution in monosomic forms, chromosome instability would lead to structural changes in chromosomes. It is commonly recognized that chromosome aberrations are generated when there are two or more monosomic chromosomes in the cell. However, alien monosomic additions could also cause chromosome instability and induce chromosome abnormalities [39,40]. Therefore, the artificial development of aneuploid plants, such as monosomic addition lines, is an effective procedure to select alien translocations [39]. However, in an unstable population, an alien chromosome in monosomic form would be eliminated rapidly (within a few generations), which results in the low frequency of chromosome translocations in progeny. On the other hand, a plant with an anomalous and unbalanced genome tends to be weak, so it is unlikely to be selected from the progeny population. These factors lead to low selection efficiency for primary translocation by using monosomic addition lines. In the present study, the manipulation of the unstable genetic population was improved. Only progeny plants with target traits were continuously selected to build the next generation population, so more alien chromosomes were retained in the population, and translocations occurred in higher frequency. The weakness that is common among plants with novel chromosome aberration can be addressed by growing these plants more carefully in the field or a greenhouse. The novel CCT line, RT855-13, was selected from the progenies of these surviving plants. The results indicated that the CCT line can be effectively selected through artificially inducing chromosome instability and careful manipulation of an unstable population.

### 3.2. Effect of the Novel 1RS.1BL Translocation on Agronomic Characters and Resistance to Stripe Rust

In the past 50 years, Hundreds of wheat cultivars with 1RS.1BL translocation were released [19]. Since the 1990s, due to the new prevalence of *Pst* virulent pathotypes and the single origin of the 1RS chromosome arm [10,18,23], the *Yr9* gene lost its resistance to *Pst* pathogens [20]. To solve this problem, several new 1RS chromosome arms of rye were transferred into the wheat genome. Because rye is a cross-pollinated plant, an abundance of variations and high genetic diversity could be found in the rye population [41]. The genetic diversity of 1RS chromosome arms could be transferred into wheat and created the genetic diversity of 1RS.1BL translocation chromosomes. Therefore, these new 1RS.1BL translocation lines showed different resistance patterns to diseases and different performances of agronomic traits. Only a few novel 1RS.1BL translocation lines showed both high resistance and excellent agronomic traits [10,20,23,42,43,44], which means that not every novel translocation line has value to the wheat-breeding program.

In the present study, two novel translocation lines, RT855-13 and RT855-14, derived from Weining rye, showed high resistance to four *Pst* isolates that are prevalent in southwestern China (Table 2). Because four *Pst* isolates were virulent to wheat parent MY11, isolates CYR29, CYR31, and SY5 were also virulent to the *Yr9* gene, and new resistance genes in RT855-13 and RT855-14 must be located on the 1RS. These new resistance genes, which were derived from Weining rye, provided better resistance to *Pst* diseases than the *Yr9* gene.

In previous studies, it was often reported that the effects of 1RS.1BL translocation chromosomes on agronomic performance were different in different wheat backgrounds. Several studies reported that there were no significant effects on grain yield in 1RS.1BL translocation lines [45], and several other studies indicated that 1RS.1BL translocation lines could significantly improve grain yield and other agronomic traits [10,20,43,46]. The sources of 1RS were very important; for example, the 1RS.1BL translocation lines with better sources of 1RS could produce constantly higher grain yield [10,47]. In this study, both translocation lines showed better agronomic traits than their wheat parent MY11. Although both lines were lower in KW and TKW than MY11, both lines have significantly higher NS, leading to a significant increase in GY (Table 1).

### 3.3. Effect of the CCT on Agronomic Characters and Resistance to Stripe Rust

In human genetics, carriers with CCT usually have an unhealthy phenotype [33]. However, The CCT wheat line RT855-13 had a normal phenotype in the field, similar to that of its wheat parent and sister-line RT855-14. Both the translocation lines RT855-13 and RT855-14 harbored the same genetic material but with different translocation forms (Figure 4 and Figure 5). The genetic effects of CCT can be detected by comparing the two translocation lines. Both the translocation lines have resistance to stripe rust from transferring the 1RS chromosome arm. However, the CCT line RT855-13 retained better resistance as adult plants compared with RT855-14 (Table 2), indicating that CCT promotes resistance to stripe rust of wheat. It is also reported that the 5B/7B reciprocal translocation in wheat enhanced resistance to stripe rust [48]. The results suggested a positional effect of chromosome translocations on resistance to diseases. However, whether the enhanced resistance originated from the CCT themselves and/or promotion of CCT to resistance gene in the 1RS.1BL chromosome should be further investigated.

The introduction of the 1RS arm into wheat significantly improved several agronomic traits, such as kernel number per spike, number of spikes per unit area, harvest index, and yield capacity (Table 1). The CCT line RT855-13 exhibited taller spikes and greater numbers of spikelets per spike than RT855-14 (Table 1). In the field, line RT855-13 had more prostrate growth in the winter and a later heading stage than RT855-14. The phenotypic variation of plants with reciprocal translocation, which have no loss or gain of chromatin, is ascribed to position effects, which show mainly on quantitative traits with significant differences. There is a vernalization gene *VRN-2* in the 4BS chromosome [17], so it is reasonable that the position effects of 4B/3D reciprocal translocations on the *VRN-2* gene may have changed the growth habit of RT855-13, leading to a longer spike development stage and a later heading stage and a larger spike than its counterpart line T855-14.

### 3.4. Evolutionary Significance and Breeding Value of Novel CCT Line

Several alien translocations, such as 1RS.1BL, 1RS.1AL, and 6VS.6AL, have been widely used in wheat-breeding programs [10,12]. Some intra-species translocations exist in wheat cultivars and local varieties and may be retained because they impart favorable resistance to diseases and facilitate other adaptive advantages [5,16,17,48,49]. In the present study, the results indicated that CCT has positive effects on agronomic traits and resistance to *Pst*, which are advantageous for wheat improvement and evolutionary adaptation. However, when plants with CCT were crossed with normal relatives, the F_1_ generation is semi-sterile because of chromosome abnormality in meiosis. This phenomenon would negatively influence wheat breeding. Effective selection in wheat breeding would therefore require a larger starting population. Several cultivars of wheat that contain CCT between 1RS.1BL and 7D chromosomes have been released in northern China [50]. These wheat cultivars with CCT have been distributed widely in northern China, indicating that the CCTs are valuable resources in wheat improvement. The CCT would also be excellent materials in researches for position effects, gene expression, and chromosome evolution. However, the successful utilization of CCT is still globally rare among wheat-breeding programs because CCT resources are scarce. The creation of novel resources of CCT may be a future target in wheat improvement.

## 4. Materials and Methods

### 4.1. Development of Plant Materials with Chromosome Translocations

Mianyang11 (MY11) was a wheat cultivar released in China in 1981 and used as wheat parent and crossed with Weining rye, which was collected from southwestern China. MY11 contains the *kr1* gene and no rye chromatin. The Weining rye exhibits considerable genetic differences compared with rye accessions collected from west Asia and Europe [41]. Weining rye was used as the plant material for the rye genome sequence. A total of 39,355 genes were identified in the Weining rye genome. Among them, 1909 disease resistance-associated genes were mapped to the seven assembled chromosomes of Weining rye [51]. The F_1_ seedlings were treated with 0.05% colchicine + 3% dimethyl sulfoxide for eight hours to produce the C_1_ plants. Then the amphidiploid plants were backcrossed twice with MY11 to produce BC_2_F_1_ plants. The line 98–855 (2n = 43 = 42W + 1′1R) was selected and reproduced in an isolation field by selfing. From the BC_2_F_5_ progenies, a plant RT855 was selected that harbored a new 1RS.1BL translocation and lacked a 4B chromosome, 2n = 41 (Figure 1 and Figure 4A). However, in the progenies of RT855, several plants were recovered that contained a pair of 4B chromosomes, 2n = 42 (Figure 4B). Two novel translocations were obtained from the progeny of RT855, named RT855-13 and RT855-14, respectively. MY11 is highly susceptible to stripe rust. A 1RS.1BL cultivar, Chuan-Nong11 (CN11), which contains the *Yr9* gene, was used as the control.

### 4.2. Detection of ω-Secalin Protein

The *Sec-1* gene is located on 1RS, encodes the ω-Secalin protein, can be detected by acid polyacrylamide gel electrophoresis (A-PAGE), and is considered as a protein marker for 1RS.1BL translocations. The A-PAGE analysis of RT855-13 and RT855-14 were performed according to Ren et al. [29].

### 4.3. Molecular Analysis

Co-dominant PCR was used to identify the chromosome construction of RT855-13 and RT855-14. The seeds of RT855-13 and RT855-14 used in this experiment were collected from three different generations. Total genomic DNA was isolated from young leaves by the surfactant cetyltrimethylammonium bromide. Four primers were used together in one PCR reaction: O11B3, O11B5, ω-sec-P1, and ω-sec-P2 [27]. The PCR was performed according to Chai et al. [27] and Ren et al. [29]. PCR reactions were analyzed by electrophoresis on 1% agarose gels stained with ethidium bromide.

### 4.4. Identification of Chromosomes

RT855-13 and RT855-14 were identified by multi-color fluorescence in situ hybridization (MC-FISH) techniques. Root mitotic metaphase cells of each plant were prepared as described [52]. Oligo-pAs1-1, Oligo-pSc119.2-1, and genomic DNA of rye were used as probes in MC-FISH analysis as described by Tang et al. [28].

The clone 6c6 is a wheat-specific centromeric sequence [29], and the probe pMD-CEN-3 is specific for the rye centromeric region [53]. The structures of centromeres were identified by these two primers. The telomeres were identified by sequence CCCTAAACCCTAAACCCTAAACCCTAAA. It was used with 6c6 to identify the intact chromosomes. Labeling processes of all probes, in situ hybridization, and image acquisition and processing were performed as described [28,29,31]. Images were captured using an epifluorescence microscope (model BX51, Olympus, Center Valley, PA, USA) equipped with a cooled chargecoupled device camera and operated with the software program HCIMAGE Live (version 2.0.1.5, Hamamatsu Corp., Sewickely, PA, USA).

### 4.5. Field Experiments for Determining Agronomic Traits

Two translocation lines and their wheat parent, MY11, were planted in the growing seasons 2014–2015. All lines were planted in standard practices with irrigation in Qionglai, China. The field experiments were designed in randomized, complete-block with three replications. Each plot was 3-m long, 25-cm apart, and spaced with eight rows. The plant density was 160 seedlings per square meter. Data for yield components analysis were collected from each plot during harvest time. Ten random plants from each plot were chosen (total 30 plants) to determine PH, SLN, KN, and KW. The NS was determined by one square meter from the center rows. The GY, TKW, and HI were determined after harvest of all plots. All the grain weight data were obtained based on 12% moisture.

The grain yield estimates were performed according to Kim et al. [47] and Ren et al. [10]. Fungicide was used to control diseases.

### 4.6. Resistance Analysis

Seedlings of two translocation lines, their wheat and rye parents, and the control were inoculated in the greenhouse, with three replicated. The wheat cultivar CN11 was used as a control (*Yr9*). Plants were inoculated with two *Pst* isolates, CYR29 (virulent to *Yr1*, *2*, *3*, *8*, *9*, *19*, *23*) and CYR31 (virulent to *Yr1*, *2*, *3*, *6*, *7*, *9*, *27*), and two new emerging isolates, SY5 and SY6, that are virulent in the field to many newly cultivars [10,54]. Infection types are scored based on a 0–9 scale [54]. Infection types 0–3 are recorded as resistant, 4–6 are recorded as intermediate, and 7–9 are recorded as susceptible. The *Pst* isolates were provided by the Plant Protection Institute, Gansu Academy of Agricultural Sciences, China.

Furthermore, RT855-13, RT855-14, MY11, and CN11 were planted in the field. Screen the new translocation lines resistant to stripe rust in the field (natural infection in open field) is very important because Sichuan province is the most serious area where stripe rust occurs in China. Infection types are scored as described by Wan et al. [54].

### 4.7. Statistical Analysis

Analysis of variance was performed for each agronomic character. Data were analyzed by ANOVA, where replicates were regarded as random effects. Significantly different means were separated at the 0.05 probability level by the least significant difference test (LSD) [10]. All the analyses were performed using the SPSS software package version 21.0 (SPSS Inc., Chicago, IL, USA).

## 5. Conclusions

In this study, two wheat-rye 1RS.1BL translocation lines, RT855-13 and RT855-14, were selected and identified from the distance cross between wheat Mianyang11 and rye Weining. Both lines showed better resistance and agronomic traits than their wheat parent, Mianyang11. Besides the 1RS.1BL translocation chromosome, there are other two pairs of balanced reciprocal translocations, 3DS.4BS^DS^ and 3DL-4BS^PS^.4BL, occurring in RT855-13. The CCT line RT855-13 showed better resistance and agronomic traits than RT855-14. The results indicated that the CCT line can be effectively selected through artificially inducing chromosome instability and careful manipulation of an unstable population, and the position effects of CCT can lead to beneficial variations in multiple traits, making CCT lines a valuable genetic resource to wheat breeding programs.

## Figures and Tables

**Table 1:** Mean agronomic traits of two translocation lines, RT855-13 and RT855-14, and their wheat parent.

Translocation Lines and Wheat Parent	PH (cm)	SLN (per Spike)	KN (per Spike)	KW (per Spike)	TKW (g)	NS (m^−2^)	GY (kg/ha)	HI (%)
RT855-13 (CCT)	92.6b	24.4a	59.20a	2.15b	36.4b	362.7a	6081.1a	47.53a
RT855-14	101.3a	21.8b	52.55ab	2.02b	38.5b	350.4a	5936.9a	46.09ab
Mianyang 11	97.8a	21.4b	45.86b	2.51a	49.2a	273.2b	5428.2b	44.99b

PH, plant height; SLN, spikelet number per spike; KN, kernel number per spike; KW, kernel weight per spike; TKW, 1000-kernel weight; NS, number of spikes per square meter; GY, grain yield; HI, harvest index. Values with the same letter in the same column do not differ significantly at *p* < 0.05.

**Table 2:** Analysis of resistance to stripe rust in translocation lines RT855-13 and RT855-14 under *Puccinia striiformis* f. sp. *tritici* (*Pst*) inoculation or field natural infection.

Lines	CYR29	CYR31	SY5	SY7	In the Field
RT855-13 (CCT)	0	0	0	1	2
RT855-14	0	0	0	1	6
Mianyang 11 (wheat parent)	8	7	8	7	8
Chuan-Nong 11 (CK)	8	8	8	1	7
Weining rye	0	0	0	0	0

Disease severities are recorded on an infection type scale of 0–9 as described by Wan et al. (2004). where 0 = immune, no visible symptoms; 1–3 = resistant, increasing from 1, flecks and no necrosis to 2, necrosis to 3, chlorotic areas with slight sporulation; 4–6 = intermediate, chlorotic areas decreasing in amount, while mycelium and conidial production increases from slight to moderate; 7–9 = susceptible, increasing amount, size, and density of mycelium and conidia to a fully compatible reaction.

## Data Availability

Data is contained within the article.
